# Tracking organelle activities through efficient and stable root genetic transformation system in woody plants

**DOI:** 10.1093/hr/uhad262

**Published:** 2023-11-26

**Authors:** Jinli Gong, Yishan Chen, Yanna Xu, Miaofeng Gu, Haijie Ma, Xiaoli Hu, Xiaolong Li, Chen Jiao, Xuepeng Sun

**Affiliations:** Collaborative Innovation Center for Efficient and Green Production of Agriculture in Mountainous Areas of Zhejiang Province, College of Horticulture Science, Zhejiang A&F University, Hangzhou 311300, Zhejiang, China; Key Laboratory of Quality and Safety Control for Subtropical Fruit and Vegetable, Ministry of Agriculture and Rural Affairs, Zhejiang A&F University, Hangzhou 311300, Zhejiang, China; Collaborative Innovation Center for Efficient and Green Production of Agriculture in Mountainous Areas of Zhejiang Province, College of Horticulture Science, Zhejiang A&F University, Hangzhou 311300, Zhejiang, China; Key Laboratory of Quality and Safety Control for Subtropical Fruit and Vegetable, Ministry of Agriculture and Rural Affairs, Zhejiang A&F University, Hangzhou 311300, Zhejiang, China; Collaborative Innovation Center for Efficient and Green Production of Agriculture in Mountainous Areas of Zhejiang Province, College of Horticulture Science, Zhejiang A&F University, Hangzhou 311300, Zhejiang, China; Key Laboratory of Quality and Safety Control for Subtropical Fruit and Vegetable, Ministry of Agriculture and Rural Affairs, Zhejiang A&F University, Hangzhou 311300, Zhejiang, China; Collaborative Innovation Center for Efficient and Green Production of Agriculture in Mountainous Areas of Zhejiang Province, College of Horticulture Science, Zhejiang A&F University, Hangzhou 311300, Zhejiang, China; Key Laboratory of Quality and Safety Control for Subtropical Fruit and Vegetable, Ministry of Agriculture and Rural Affairs, Zhejiang A&F University, Hangzhou 311300, Zhejiang, China; Collaborative Innovation Center for Efficient and Green Production of Agriculture in Mountainous Areas of Zhejiang Province, College of Horticulture Science, Zhejiang A&F University, Hangzhou 311300, Zhejiang, China; Key Laboratory of Quality and Safety Control for Subtropical Fruit and Vegetable, Ministry of Agriculture and Rural Affairs, Zhejiang A&F University, Hangzhou 311300, Zhejiang, China; Collaborative Innovation Center for Efficient and Green Production of Agriculture in Mountainous Areas of Zhejiang Province, College of Horticulture Science, Zhejiang A&F University, Hangzhou 311300, Zhejiang, China; Key Laboratory of Quality and Safety Control for Subtropical Fruit and Vegetable, Ministry of Agriculture and Rural Affairs, Zhejiang A&F University, Hangzhou 311300, Zhejiang, China; Collaborative Innovation Center for Efficient and Green Production of Agriculture in Mountainous Areas of Zhejiang Province, College of Horticulture Science, Zhejiang A&F University, Hangzhou 311300, Zhejiang, China; Key Laboratory of Quality and Safety Control for Subtropical Fruit and Vegetable, Ministry of Agriculture and Rural Affairs, Zhejiang A&F University, Hangzhou 311300, Zhejiang, China; Institute of Biotechnology, Zhejiang University, Hangzhou 310058, Zhejiang, China; Collaborative Innovation Center for Efficient and Green Production of Agriculture in Mountainous Areas of Zhejiang Province, College of Horticulture Science, Zhejiang A&F University, Hangzhou 311300, Zhejiang, China; Key Laboratory of Quality and Safety Control for Subtropical Fruit and Vegetable, Ministry of Agriculture and Rural Affairs, Zhejiang A&F University, Hangzhou 311300, Zhejiang, China

## Abstract

Due to the protracted transgenic timeline and low efficiency in stable genetic transformation of woody plants, there has been limited exploration of real-time organelle imaging within stable transgenic woody plant cells. Here, we established an efficient *in vivo* genetic transformation system for woody plants using an *Agrobacterium rhizogenes*-mediated approach. This system was successfully validated in multiple perennial woody species. Using citrus as a model, we introduced organelle-targeted fluorescent reporters via genetic transformation and investigated their subcellular localization and dynamics using advanced imaging techniques, such as confocal microscopy and live-cell imaging. Moreover, we subjected transgenic MT-GFP-labeled mitochondria in root cells to stress conditions simulating agricultural adversities faced by fruit crops. The stress-induced experiments revealed notable alterations in mitochondrial morphology. Our study contributes novel insights into membrane trafficking processes, protein localization dynamics, and cellular physiology in woody plants, while also providing stable and efficient genetic transformation methods for perennial woody species.

## Introduction

Biological studies have entered an era in which biological phenomena are reduced to the behavior of molecules. However, discrete biological functions are rarely dependent on individual molecules. Rather, they frequently emerge as a result of intricate cellular activities working in concert. Therefore, comprehending the behavior and interactions of cellular components across diverse developmental stages and environmental conditions holds utmost significance in addressing fundamental biological questions. This is particularly remarkable for fruit plants as the development and ripening of fruit are tightly associated with the morphological and physiological changes of organelles [1]. Unfortunately, most research on plant cell biology has been accomplished in model systems, leaving us with limited knowledge about the activity and dynamics of cellular components in perennial woody plants. Furthermore, many studies in cell biology rely on techniques, such as mechanical disruption, sectioning, or enzymatic degradation, to circumvent plant cell walls. Consequently, conclusions have been extrapolated from examinations of preserved plant tissues, which may not always reflect the authentic *in vivo* conditions within plant cells [2].

To investigate the dynamics of intracellular compartments in fruit perennials, we have developed a transient transformation approach in citrus fruit, which can be used to monitor subcellular activities *in vivo* [2]. However, its efficiency might vary across fruit varieties. Conversely, the application of a robust genetic transformation system, such as the one mediated by *Agrobacterium tumefaciens*, has demonstrated extensive utility in probing gene functionality and genetic modifications in model plants and crops [3–5], but its adoption has been hindered in woody plants due to their extended growth cycles and inherently low transformation efficiency [4, 6, 7]. In fact, among the extensive assemblage of over 370 000 higher plant species populating the world, a mere fraction—less than 0.1%—have proven successful for genetic modifications, primarily due to the constrains inherent within the genetic transformation system [8]. Even for those plants amenable to genetic modification, the current transgenic methods rely on laborious and costly tissue culture procedures, and often fall short of desired outcomes [8, 9]. Hence, establishment of a stable, rapid, and efficient transgenic system for woody plants is of particular importance. *Agrobacterium rhizogenes*, a close relative of *A. tumefaciens*, can induce adventitious roots, known as ‘hairy roots’, when the leaves or stems of plants are injured and infected [10]. Upon infection, *A. rhizogenes* transfers its Ri plasmid into host nuclei, by which the hairy roots can be genetically modified. The root genetic transformation system mediated by *A. rhizogenes* involves a simple and rapid procedure, which avoids tissue culture and has been widely employed for transgenic studies in various plant species over the past decade [11–15]. Although the hairy root system has proven valuable for studying gene function, its utility in tracking organelle activities within plant cells has been largely unexplored.

In this study, we aimed to develop an *in vivo* organelle tracking system in woody plants through root transformation of various fluorescent protein markers. We have tested this approach successfully in several perennials. Subsequently, using citrus as a model, we show that mitochondria are subject to morphology changes under various stresses. In conclusion, our results not only provide an effective method for cellular biology observations in recalcitrant woody plants but also show valuable insights into the cellular responses of woody crops to extreme environmental conditions.

## Results and discussion

### *A. rhizogenes*-mediated root transformation in woody plants

**Figure 1:**
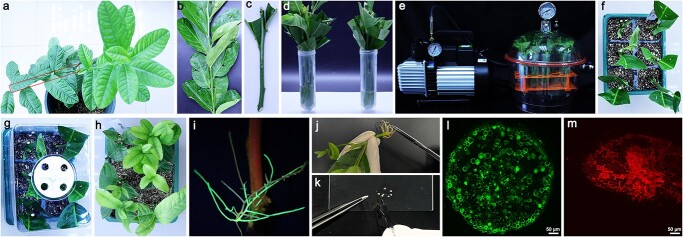
*Agrobacterium rhizogenes* K599 mediated efficient root genetic transformation. **a** Image of citron plant used for infection. Box indicates semi-lignified branches for subsequent experiment. **b** Cut fresh semi-lignified branches. **c** Branches with a length of 5–10 cm. Excess leaves were removed and the apical two leaves were pruned to one-third of their original length. **d** Branches were infiltrated in *A. rhizogenes* with an OD600 of 0.8–1. **e** Vacuum infiltration of the branches in 0.1 MPa vacuum for 30 min. **f** Infected branches were inserted into moist sterilized vermiculite and covered (**g**) to maintain humidity and facilitate rooting. **h**–**i** Growth status of aboveground and underground parts of the plants after one month of infection. **j**–**k** Dissected sections expressing fluorescent fusion proteins. **l**–**m** Laser confocal images showing GFP and RFP fluorescence of a cross-sectioned transgenic root and a fine root, respectively.

The successful genetic transformation of roots was initially evaluated in citron (*Citrus medica* L.) (Fig. 1a), an ancient and original citrus species known for its high survival rate when propagated from cuttings in vermiculite [13, 16]. In our transformation experiments, semi-lignified shoots (Fig. 1b), typically one to two years old, were carefully sectioned into segments measuring 5–10 cm in length, with a significant removal of leaves (Fig. 1c). We performed vacuum infiltration using *A. rhizogenes* strain K599, which carried a fluorescent expression vector driven by the 35S promoter (Fig. 1d and e). Following infiltration, the shoots were placed in moist vermiculite within a growth chamber under controlled conditions (26°C, 16 hours of light/8 hours of darkness) (Fig. 1f and g). After approximately two weeks, shoot buds emerged from the aboveground portion, while a substantial amount of callus formed at the wound site on the belowground branch. Within one month, as depicted in Fig. 1h and i, these newly formed buds rapidly developed into leaves. Simultaneously, a significant number of hairy roots emerged from the injured area. To distinguish transgenic and non-transgenic roots, a fluorescence protein-excited light was used. Successful transgenic roots showed strong green fluorescence, while non-transgenic roots did not (Fig. 1i). We cross-sectioned the roots into thin slices and transferred them onto glass slides for laser confocal microscopic observation (Fig. 1j–l). Alternatively, for more delicate root structures, the process of sectioning could be omitted, allowing for direct visualization of root characteristics (Fig. 1m). Our observations revealed a consistent and robust distribution of fluorescence throughout the root tissues, thus demonstrating the suitability of transgenic hairy roots for the study of subcellular activities and protein localization in living cells.

To evaluate the versatility of organelle markers applicable in this system, we infected citron roots with plasmids carrying fusion protein of GFP or RFP paired with various organelle markers, including an endoplasmic reticulum (ER) marker (i.e., GFP-HDEL), an actin marker (GFP-Lifeact), a mitochondrion marker (MT-GFP), and a plastid marker (PT-RFP). Remarkably, we observed a consistently high transformation efficiency for all of these markers, resulting in robust fluorescence in all transgenic roots (Fig. S1, see online supplementary material).

**Figure 2:**
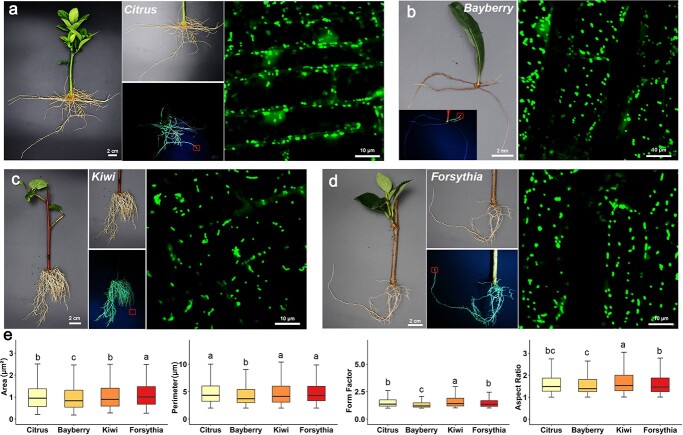
*Agrobacterium rhizogenes*-mediated root transformation of MT-GFP plasmid in citrus (**a**), bayberry (**b**), kiwifruit (**c**), and forsythia (**d**). **e** Quantification of mitochondrial morphology in the four species. Different letters indicate statistical significance (*P* < 0.05). Boxes in the panels **a**–**d** indicate root tips used for laser confocal observation of mitochondria.

We extended the application of the *A. rhizogenes*-mediated root transformation system to a diverse range of woody plants, including citrus, bayberry, kiwifruit, and forsythia, to assess its adaptability across varying species (Fig. 2). Using the plasmid carrying the MT-GFP fusion protein, we demonstrated a consistently high transformation efficiency in all four species, accompanied by clear florescence signals from the MT-GFP-labeled mitochondria (Fig. 2a–d). Statistical analysis of mitochondrial fluorescence signals indicated varied differences in mitochondrial morphology, including area, perimeter, form factor (representing roundness that was measured by perimeter^2^/4π × area), and aspect ratio, among the root cells of the four species (Fig. 2e). Kiwifruit displayed higher form factor and aspect ratio compared to the other three species, which were also visually evident in our imaging. Specifically, in root cells of citrus, bayberry, and forsythia, the majority exhibited globular mitochondria (Fig. 2a, b and d), whereas kiwifruit root cells frequently displayed tubular mitochondria (Fig. 2c). Kiwifruit also showed more pronounced branch junctions and longer branches in its mitochondrial network connectivity compared to the other three species (Fig. S2, see online supplementary material). These results demonstrate the adaptability of the *A. rhizogenes*-mediated transformation method to all four species examined. However, it is important to note that bayberry presents challenges in genetic transformation when infiltrated with *Agrobacterium* via stem cuttings. This challenge arises from the inherent difficulty of bayberry stem cuttings to initiate root formation, leading to significantly reduced plant survival rates. Instead, our proposed method involves the use of bayberry leaves, from which adventitious roots develop in the petiole region (Fig. 2b). This approach enhances the survival rates in comparison to using stem cuttings, which provides a promising strategy for genetic transformation in other recalcitrant woody plants.

Transformation of perennial woody species using *A. tumefaciens*-mediated methods is a time-consuming process. Various factors, including bacterial genotype, explant type, hormone ratio, and infection condition, can influence the transformation efficiency [17]. Hence, achieving a satisfactory transformation efficiency for perennial woody plants can be quite challenging. In contrast, *A. rhizogenes*-mediated transformation system offers a shorter cycle and higher efficiency, and has been progressively adopted in numerous plants [18, 19]. In our experiments, a large number of hairy roots that stably express recombinant plasmids can be induced by infecting branches or leaves of living plants (Figs 1 and2). Traditionally, *A. rhizogenes*-mediated transformation was primarily employed for root transformation, yielding transgenic roots rather than whole transgenic plants [20, 21]. However, in recent years, researchers have harnessed *A. rhizogenes*-induced hairy roots for plantlet regeneration, resulting in the production of stable and genetically modified plants in several species including petunias [22], *Catharanthus roseus* [23], buckwheat [24], and citrus [25]. This indicates the substantial potential for generating stable genetically modified plants through *A. rhizogenes*-mediated transformation in a broad range of woody plants.

### Live cell imaging of citrus root cells

**Figure 3:**
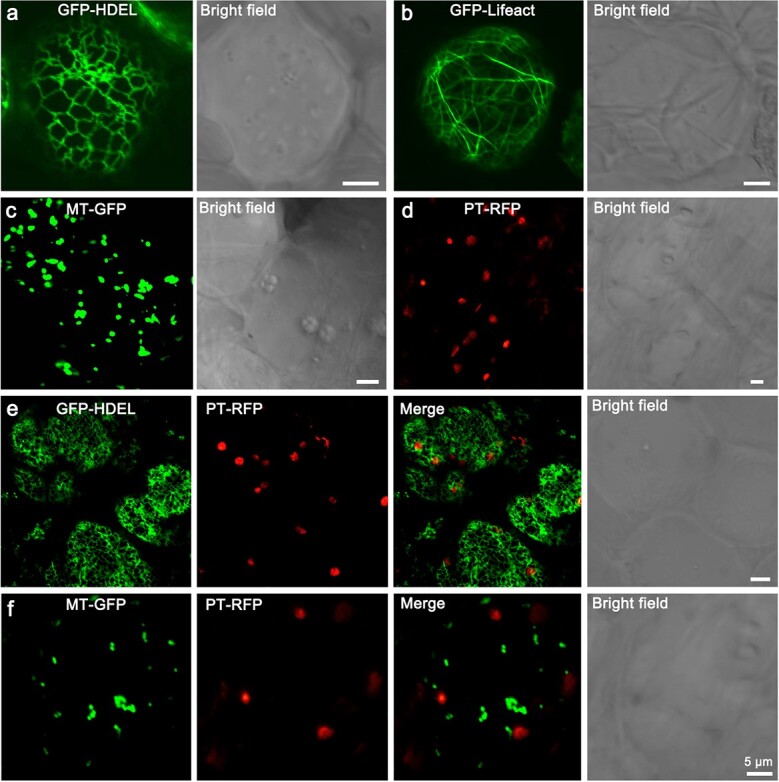
Subcellular localization of various fluorescent fusion proteins in citrus root cells. **a**–**d** Representative images of fluorescent protein fusions localized to ER (GFP-HDEL), actin cytoskeleton (GFP-Lifeact), mitochondria (MT-GFP), and plastids (PT-RFP). **e–f** Co-expression of GFP-HDEL/MT-GFP and PT-RFP identified the close association between the ER network/ mitochondria and plastids in citrus root cells. Scale bar, 5 μm.

Determining the subcellular localization of a protein is a fundamental prerequisite in understanding its function [26]. Currently, investigation of subcellular protein localization in most crops primarily relies on heterologous transient expression systems, such as onion epidermal cells, *Arabidopsis* leaf protoplasts, rice leaf protoplasts, or tobacco leaves. However, due to the potentially limited conservation in protein sequences and regulatory factors governing protein trafficking within these heterologous expression systems, there is a risk of engendering spurious subcellular localization results in such approaches [27–29]. In this study, we infiltrated various organelle markers labeled with GFP or RFP, including those for ER (Fig. 3a), actin cytoskeleton (Fig. 3b), mitochondria (Fig. 3c), and plastid (Fig. 3d). Confocal microscopy revealed that protein localization in citrus root cells was consistent with those in *N. benthamiana* leaf epidermal cells (Fig. S3, see online supplementary material). It is essential to emphasize the importance of minimizing mechanical damage and external pressure during tissue sectioning or imaging, especially when examining the delicate structures of the ER and actin cytoskeleton, as they are susceptible to mechanical disruption [2]. Subsequently, we co-infiltrated different markers along with fusion proteins of GFP and RFP to explore potential connections or co-localization between organelles. For instance, co-infiltration of GFP-HDEL and PT-RFP demonstrated the physical association between the ER and plastids in plant cells (Fig. 3e). This close relationship and the various interactions between ER and plastids have been well-documented [30]. The ER membrane extends around plastids, forming contact sites known as plastid-ER contact sites or plastid-associated ER (pER). These contact sites facilitate the communication and exchange of lipids, ions, and proteins between the ER and plastids [31, 32]. Their mutual interaction plays a pivotal role in protein trafficking, lipid transfer, and chloroplast biogenesis [33]. Furthermore, we co-expressed MT-GFP and PT-RFP (Fig. 3f) to investigate communication between mitochondria and plastids in citrus root cells. To enhance the visualization of labeled chloroplasts (PT-RFP), we selected green roots exposed to luminal illumination for microscopic observation. The results showed a notable alteration in mitochondrial morphology, wherein the spheroid mitochondria metamorphosed into filamentous or dot-like structures (Fig. 3f), suggesting an association between mitochondrial morphology and the light-induced cellular energy demands. Mitochondria and plastids cooperate and exchange metabolites to support cellular energy production, metabolic pathways, and organelle functions. This interconnectedness is crucial for the overall cellular homeostasis and proper functioning of plant cells [34].

**Figure 4:**
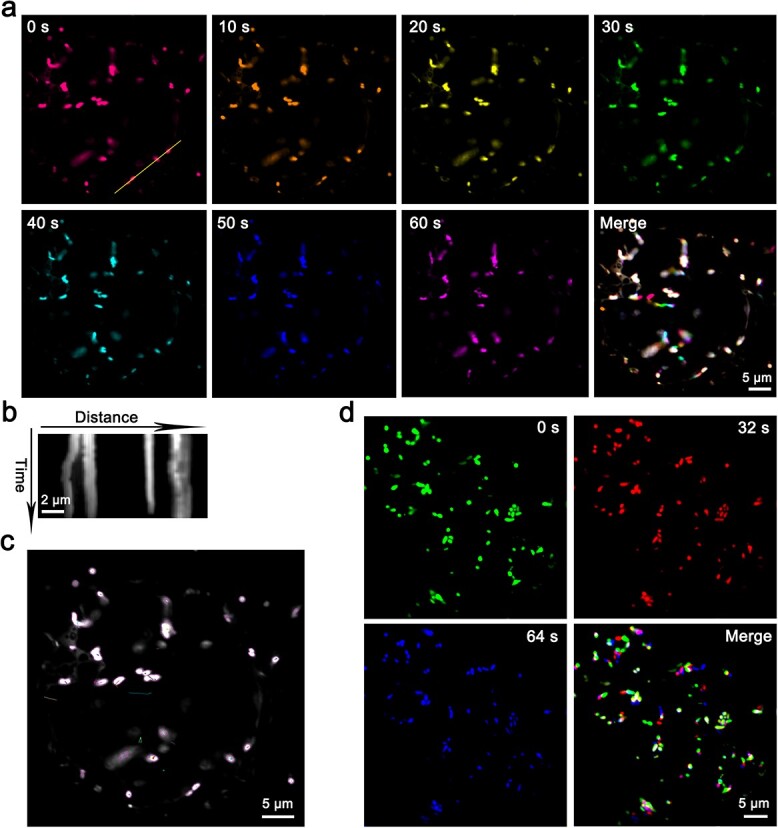
Live cell imaging of MT-GFP reveals the dynamic movement of the mitochondria in citrus root cells. **a** Time series of MT-GFP-labeled mitochondria over 60 s. The images representing the different time points are pseudocolored. The merged picture with separated signals indicates that the puncta are mobile. **b** A kymograph was generated along the line, demonstrating the slight movement of these puncta. **c** Automated mitochondria-particle tracking with TrackMate. **d** Time-lapse images of MT-GFP-labeled mitochondria over 64 s in another root cells. Scale bar, 5 μm.

The dynamic processes of mitochondrial movement and morphological regulation play a crucial role in the cellular energy supply, metabolic regulation, and normal physiological functions [35, 36]. Aberrant mitochondrial movement and morphological regulation have been associated with various diseases and physiological abnormalities. The study of mitochondrial dynamics and morphology is therefore of vital biological importance. Through our observation of the movement trajectories of mitochondria labeled with MT-GFP, the results showed that movement is diverse and multidirectional, exhibiting randomness and complexity, as indicated by our time-series images, kymograph, and tracking analysis (Fig. 4).

**Figure 5:**
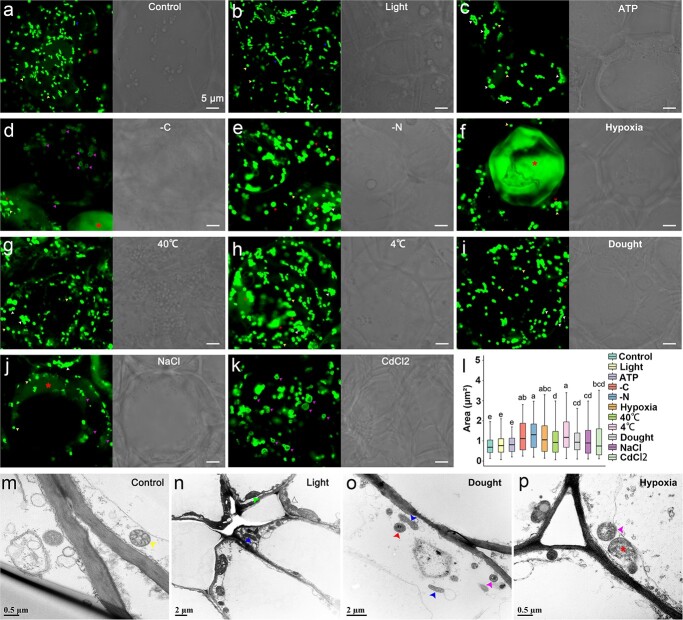
Laser confocal shows the morphology of mitochondria in different situations (**a**–**k**), 2-D statistics of area (**l**) and ultrastructural observations of mitochondria by TEM (**m**–**p**). Red arrows, swelling mitochondria; yellow arrows, globular mitochondria; blue arrows, elongated mitochondria; violet arrows, vacuolated mitochondria; white arrows, mitochondrial aggregate; red asterisks, bursting mitochondria; green arrows, chloroplasts. Scale bar, 5 μm. Different letters in panel **l** indicate statistical significance (*P* < 0.05).

### MT-derived fluorescence imaging demonstrates mitochondrial alterations under various stresses

Mitochondria, the vital organelles associated with life and respiration, often represent the final remnants of vitality. As such, mitochondria can act as discerning indicators, gauging the degree of stress plants encounter amidst adverse conditions [37]. We subjected transgenic roots carrying MT-GFP to various treatments, including nutrient deficiency (-C, -N), high temperature (40°C), low temperature (4°C), salt stress (NaCl), and heavy metal stress (CdCl_2_), to simulate the stresses that fruit crops may encounter in their natural environment (Fig. 5). In normal root cells, mitochondria were uniformly distributed throughout the cytoplasm, with a substantial population of globular mitochondria engaged in erratic motion (Fig. 5a; movie S1, see online supplementary material); and transmission electron microscopy (TEM) revealed intact mitochondrial structures with a dense matrix and random arrangement of cristae structure (Fig. 5m). Under light exposure, elongated and interconnected mitochondria appeared in root cells (Fig. 5b and n). Supply of ATP led to mitochondrial aggregation (Fig. 5c) and a reduction in their number (Fig. S4, see online supplementary material). When exposed to 40°C, mitochondria enlarged and swelled, leading to an increase in their area (Fig. 5g and l), suggesting an increased occurrence of mitochondrial fusion. Conversely, at low temperature (4°C), mitochondria continued to enlarge and presented ER structures (Fig. 5h and l), suggesting that 4°C cold stress might inflict more severe damage on citrus root cells compared to the 40°C heat stress. Under nitrogen-deficient conditions, mitochondria maintain relatively high activity, suggesting frequent mitochondrial fission and fusion, as evidenced by an increase in both the number and area of mitochondria (Fig. 5e and l; Fig. S4, see online supplementary material). Nonetheless, carbon deficiency seemed to cause significant damage to mitochondria, as most of them displayed weak fluorescence, became swollen, formed circular vacuoles, accompanied by a noticeable decrease in mitochondrial abundance and an elevated form factor (Fig. 5d; Figs S4 andS5, see online supplementary material). A similar situation occurred under heavy metal Cd^2+^ stress, where the generation of numerous vacuoles indicated disruption of mitochondrial cristae structure, and scattered mitochondrial fluorescence was observed throughout the cytoplasm (Fig. 5k). During NaCl treatment, the appearance of vacuolar structures and the widespread distribution of extensive fluorescent signals in the cytoplasm were observed, concomitant with a reduction in mitochondrial number (Fig. 5j; Fig. S4, see online supplementary material). The drought stress induced by 20% PEG6000 led to differentiated mitochondrial morphology, as evidenced by swollen, vacuolated or elongated mitochondria (Fig. 5i and o), suggesting the occurrence of frequent mitochondrial fission and fusion. Severe mitochondrial damage occurred under oxygen deprivation stress, with mitochondria swelling and some eventually rupturing, leading to widespread mitochondrial fluorescence in the cytoplasm and severe impediment of their movement (Fig. 5f). This observation was consistent with our TEM result, which demonstrated ruptured mitochondrial membrane and fragmented cristae, indicating the complete degeneration of mitochondria (Fig. 5p). These findings indicate that mitochondrial morphology and ultrastructure of citrus were highly responsive to hypoxia, which is consistent with previous studies [38]. Considering that mitochondrial hypoxia may be related to insufficient energy supply, we supplemented 1 mM ATP into citrus root cultures, and the results indeed demonstrated a significant alleviation of mitochondrial swelling (Fig. S6, see online supplementary material).

Mitochondria have the ability to perceive various stressors and respond accordingly. Apart from relying on fusion-fission dynamics, which help dilute and segregate damaged mitochondria, their motility and interactions with other organelles, such as ER, also play a crucial role in cellular adaptation to stress processes [39]. During our extensive observations of mitochondrial morphology under stress treatments, we occasionally observed fluorescence signals of MT-GFP colocalizing with structures resembling the ER, as seen in salt and Cd^2+^ stress (Fig. S7, see online supplementary material). To validate the result, we co-infected *Agrobacterium* carrying GFP-HEDL-labeled ER and MT-RFP-labeled mitochondria in *N. benthamiana* leaf. The result showed that mitochondrial puncta exhibited remarkable co-localization with ER contact points under salt or Cd^2+^ treatments (Fig. S8, see online supplementary material). Compared to GFP-HEDL-labeled ER and MT-RFP-labeled mitochondria observed in normally growing tobacco leaf cells (movie S2, see online supplementary material), exposure to salt resulted in the enlargement of mitochondrial puncta, accompanied by apparent mitochondria moving along the ER (movie S3, see online supplementary material).

**Figure 6:**
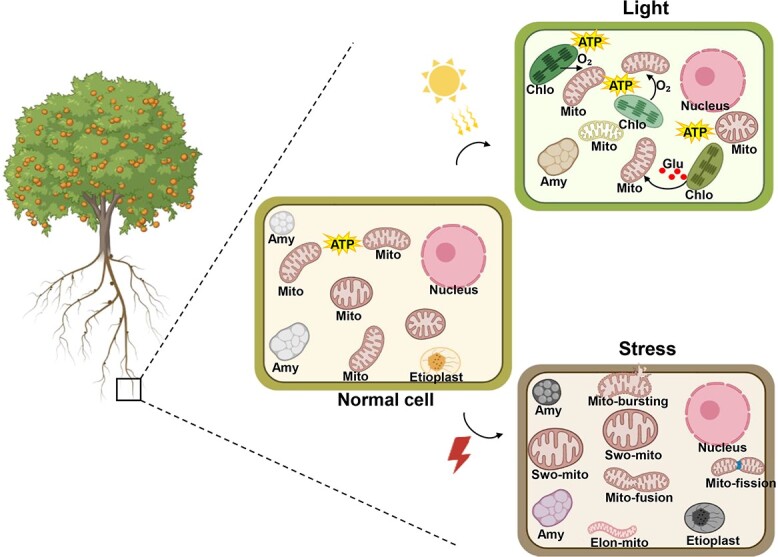
Model of mitochondrial morphology in citrus root cells under different conditions. ATP, adenosine triphosphate; Chlo, chloroplast; Mito, mitochondria; Amy, amyloplast; Glu, glucose, Mito-bursting, mitochondria bursting; Swo-mito, swollen mitochondria; Elog-mito, elongated mitochondria; Mito-fusion, mitochondria fusion; Mito-fission, mitochondria fission.

Mitochondria are among the most sensitive organelles to various damage [40]. Our results show that mitochondria in different conditions undergo remarkable changes (Fig. 6), primarily manifested by alterations in mitochondrial count, size, and structure. Under mild stress conditions, mitochondria exhibit swelling, and frequent occurrences of mitochondrial fission and fusion within the cell. However, severe stress leads to irreversible mitochondrial bursting. In conclusion, we performed subcellular-level investigations on mitochondrial morphology and dynamics in root cells of woody plants using fluorescent organelle markers, and observed the behavior of mitochondria under a series of stress conditions. Future studies using this method could potentially be useful to reveal the functions and regulatory mechanisms of a particular organelle, as well as shedding light on the interplay among organelles in woody plants.

## Materials and methods

### Plant material and growth conditions

The branches of the citron (*C. medica* L.), *Actinidia valvata* Dunn, and *Myrica rubra cv.* ‘Dongkui’ were all pruned as needed from the germplasm resource nursery of Zhejiang A&F University, while the forsythia branches came from an outdoor garden of the university. All the plants were cultivated in the open under natural conditions, and the branches were pruned as they were used.

After transformation, the branches were placed in a controlled environment chamber, maintaining a temperature of 26°C and a light cycle of 16 hours of illumination followed by 8 hours of darkness, while ensuring a consistently moist vermiculite throughout the rooting process.

### *Agrobacterium*-infiltrated hairy root transformation

Healthy and semi-lignified branches were selected for agro-infiltration using the following infiltration procedure. *A. rhizogenes* strains carrying the recombinant fluorescent plasmids were cultured in liquid LB medium supplemented with appropriate antibiotics overnight at 28°C, to an O.D. (optical density at 600 nm) of 0.8–1.0. The cultures were centrifuged at 6000 *g* for 10 min at room temperature and then resuspended in infiltration buffer (0.05 M MES, 2 mM Na_3_PO_4_, 0.5% (w/v) D-glucose, and 0.1 mM acetosyringone) to a final O.D. of 0.8. Branches were collected from the orchard and cut into small segments of 5 ~ 10 cm in length with sterilized pruning shears, ensuring that the cut surfaces are smooth. Excess leaves were removed and the top two leaves of the branch were pruned to 1/3 of their original length (as shown in Fig. 1c). Then, the base of the stems was immersed into the *A. rhizogenes* suspension mentioned above, and vacuum infiltrated for about 30 min under standard vacuum conditions. Subsequently, the branches are inserted into sterilized vermiculite and placed in the greenhouse for cultivation, maintaining a temperature of 26°C with a light cycle of 16 hours of illumination followed by 8 hours of darkness. Approximately 2–4 weeks after agro-infiltration, the success of genetic transformation was assessed by detecting the fluorescence of hairy roots using a portable excitation lamp (Luyor-3415RG, Shanghai, China).

### Confocal microscopy

The successfully transformed root tissues were delicately cut off using surgical scissors and gently placed on a microscope slide dipped in water droplets. A disposable sharp blade was swiftly used to deftly section the transgenic tissue on the microscope slide, making them as thin as possible. The slide was then covered with a cover slip for direct observation. Notably, some slender roots could be exempted from sectioning and directly subjected to microscopic imaging. An Olympus U-HGLGPS fluorescence microscope was used to conduct fluorescence imaging experiments. GFP were excited at 488 nm and detected at 500–540 nm, and RFP were excited at 561 nm and detected at 570–620 nm, respectively.

### Mitochondrial analysis

Mitochondrial tracking was achieved using Kymograph analysis [41] and Tracking [42] functionalities within the ImageJ software (https://imagej.nih.gov/ij/). For cell tracking, the thresholding detector and simple LAP tracker of TrackMate were used. The plugin of the Fiji software (https://imagej.net/software/fiji/downloads), Mitochondria Analyzer, was used for mitochondrial morphology analysis [43, 44]. All statistical analyses were based on approximately 1000 mitochondrial puncta, and the statistical significance was calculated using the Duncan test (*α* = 0.05).

### Treatments of citrus roots

Healthy and uniform sub-cultured citrus plants were selected for subsequent treatments, and a thorough cleansing of the roots was performed to remove any soil clumps. The 1/2 MS base salts with vitamins (COOlaber, PM1061) and proportionally sucrose was used for control treatments, and roots were fixed in this 1/2 MS liquid media for one week at 26°C (Control); for the light treatment (Light), the roots were cultured in the same 1/2 MS liquid medium, and exposed to an illuminance of 10 000 lux following 14-hour light/8-hour dark cycle for one week; for the ATP treatment, 1 mM ATP was introduced into the 1/2 MS liquid media; the carbon-depletion treatment (-C) followed the same procedure as the control, except for the addition of sucrose; for nitrogen deprivation (-N), citrus roots were submerged in the medium of 1/2 MS base salts -N (COOlaber, PM1061-N), with appropriate sucrose; for anaerobic stress (hypoxia), after immobilizing the citrus roots in 1/2 MS liquid media for a week, the entire plant was transferred to a sealed chamber to undergo vacuum evacuation. The vacuum was sustained for 6 hours before confocal observation. Citrus root tissues submerged in 1/2 MS liquid media were placed in the incubator at 40°C [45] / 4°C [46] for one week to simulate high/low temperature stress, respectively; 20% PEG6000 (w/v) was added to the 1/2 MS liquid medium for one week to mimic drought stress; 250 mM NaCl and 5 μM CdCl_2_ [47] were introduced into the 1/2 MS liquid medium for one week to analogue the stress of salt and heavy metal ion Cd^2+^, respectively. Three independent citrus roots were selected for microscopic imaging for all the above treatments.

### TEM

Fresh citrus root samples were cut into 1 × 3 × 1 mm^3^ pieces and immediately immersed into 2.5% glutaraldehyde. Subsequently, a vacuum treatment was applied to facilitate the sinking of the samples to the bottom of centrifuge tubes, where they were stored overnight at 4°C to ensure proper fixation. TEM was performed as described previously [48], and the samples were imaged using a Hitachi H-7650 TEM (Hitachi Ltd, Tokyo, Japan).

## Supplementary Material

Web_Material_uhad262

## Data Availability

All data are incorporated into the article and its online supplementary material.
