# Supplementary material for: Tracking organelle activities through efficient and stable root genetic transformation system in woody plants
Source: Hortic Res. 2023 Nov 26;11(1):uhad262. doi: 10.1093/hr/uhad262 (PMC10831326; doi:10.1093/hr/uhad262)
Supplement: Web_Material_uhad262 [file web_material_uhad262.zip › Supplementary_information20231030.docx]

Supplementary information for

**Tracking organelle activities through efficient and stable root genetic transformation system in woody plants**

Jinli Gong^1,2^, Yishan Chen^1,2^, Yannna Xu^1,2^, Miaofeng Gu^1,2^, Haijie Ma^1,2^, Xiaoli Hu^1,2^, [Xiaolong](https://www.nature.com/articles/s41438-021-00611-1#auth-Pengwei-Wang) Li^1,2^, Chen Jiao^3^, Xuepeng Sun^1,2*^

^1^Collaborative Innovation Center for Efficient and Green Production of Agriculture in Mountainous Areas of Zhejiang Province, College of Horticulture Science, Zhejiang A&F University, Hangzhou 311300, Zhejiang, China

^2^Key Laboratory of Quality and Safety Control for Subtropical Fruit and Vegetable, Ministry of Agriculture and Rural Affairs, Zhejiang A&F University, Hangzhou 311300, Zhejiang, China

^3^Institute of Biotechnology, Zhejiang University, Hangzhou 310058, Zhejiang, China

*Correspondence: Xuepeng Sun (xs57[@zafu.edu.cn](mailto:20210017@zafu.edu.cn))


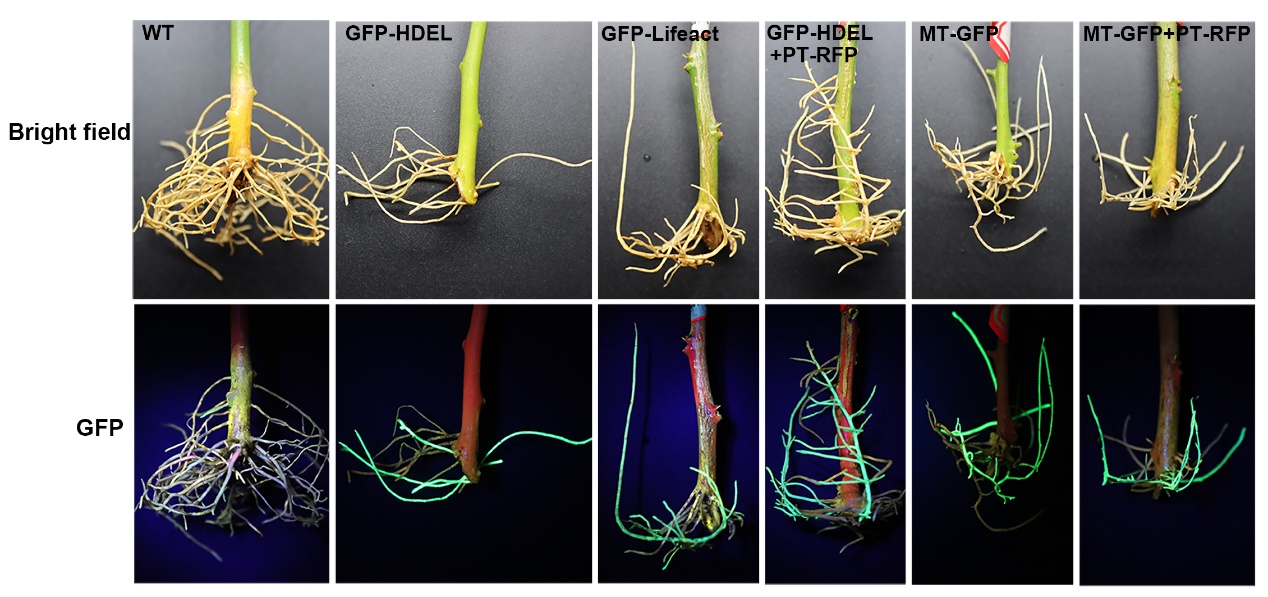


**Figure S1. Green fluorescent protein (GFP) expressed in citron roots after one month of infection.**


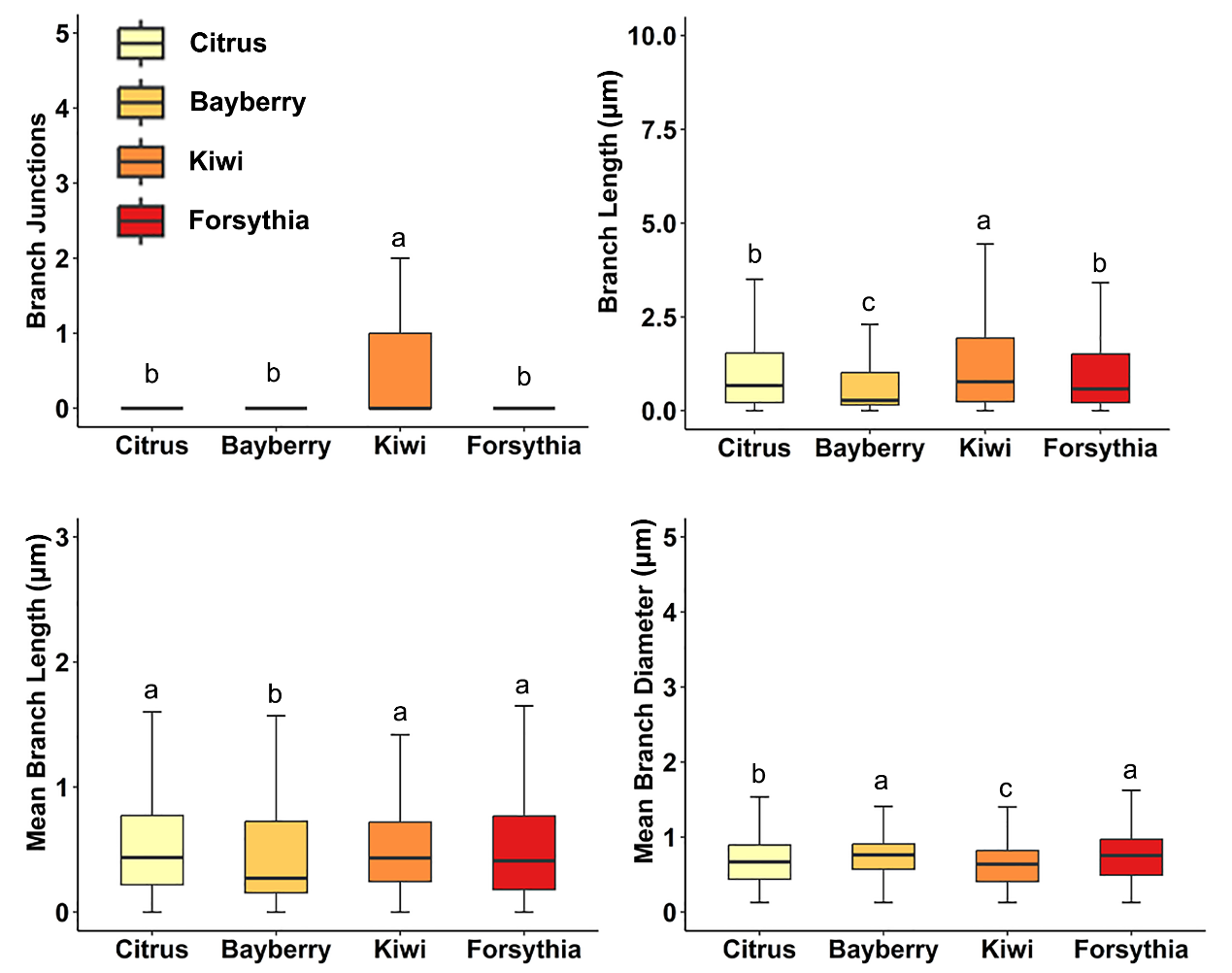


**Figure S2. Quantitative comparison of mitochondrial network connectivity in 2-dimension.** Based on visual inspection of approximately 1000 mitochondrial puncta in Figure 2. Different letters indicate statistical significance (*P* < 0.05).


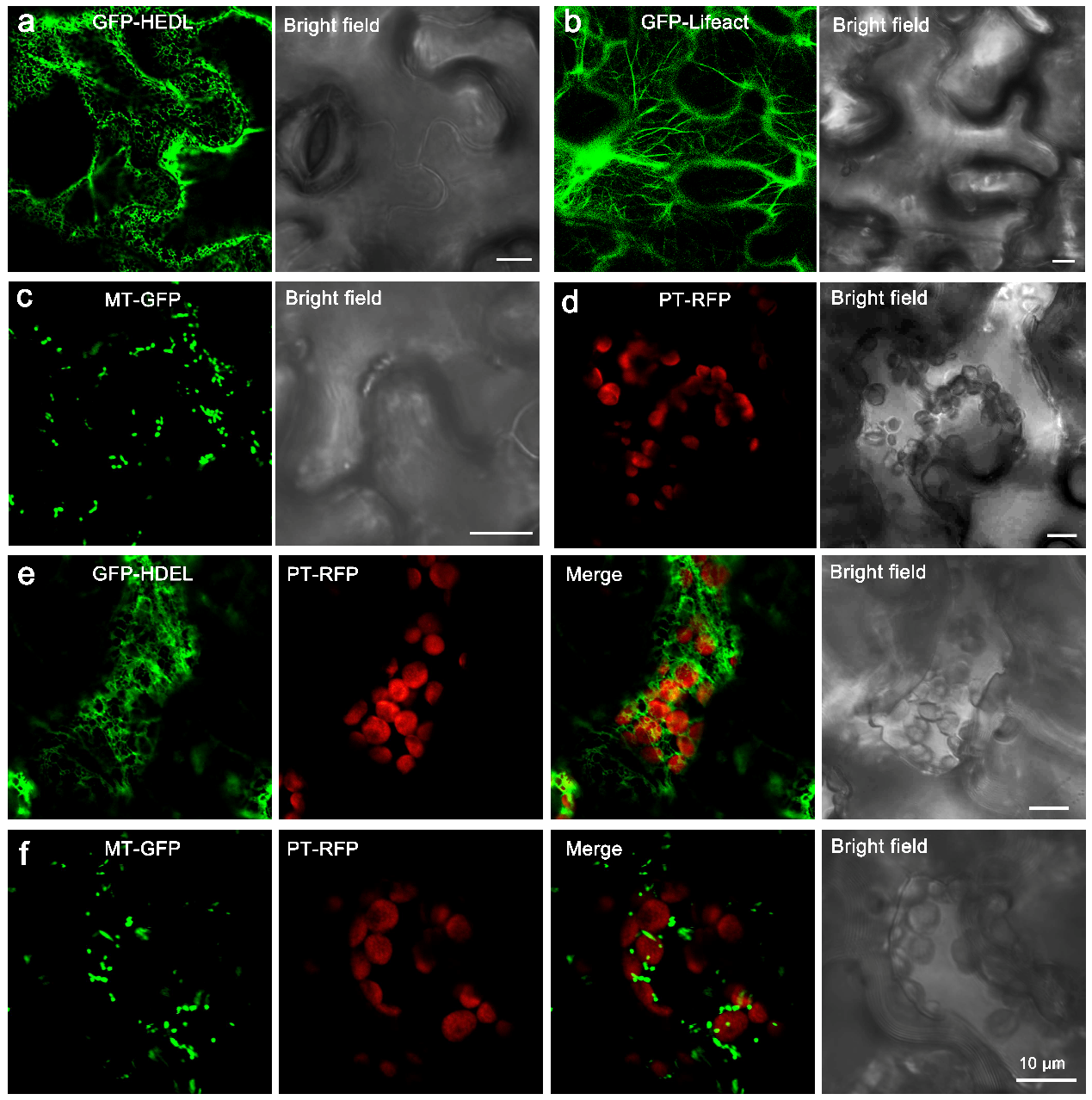


**Figure S3. Expression of fluorescent organelle markers in *N. benthamiana* leaf epidermal cells. a-d** Representative images of fluoresenct protein labelled ER (GFP-HDEL), actin cytoskeleton (GFP-Lifeact), mitochondria (MT-GFP), and plastids (PT-RFP), respectively. **e-f** GFP-HDEL/MT-GFP were co-expressed with PT-RFP in *N. benthamiana* leaf cells. respectively. All constructs were infiltrated at OD600 = 0.1, and images were taken 3 days after infiltration. Scale bar, 10 μm.


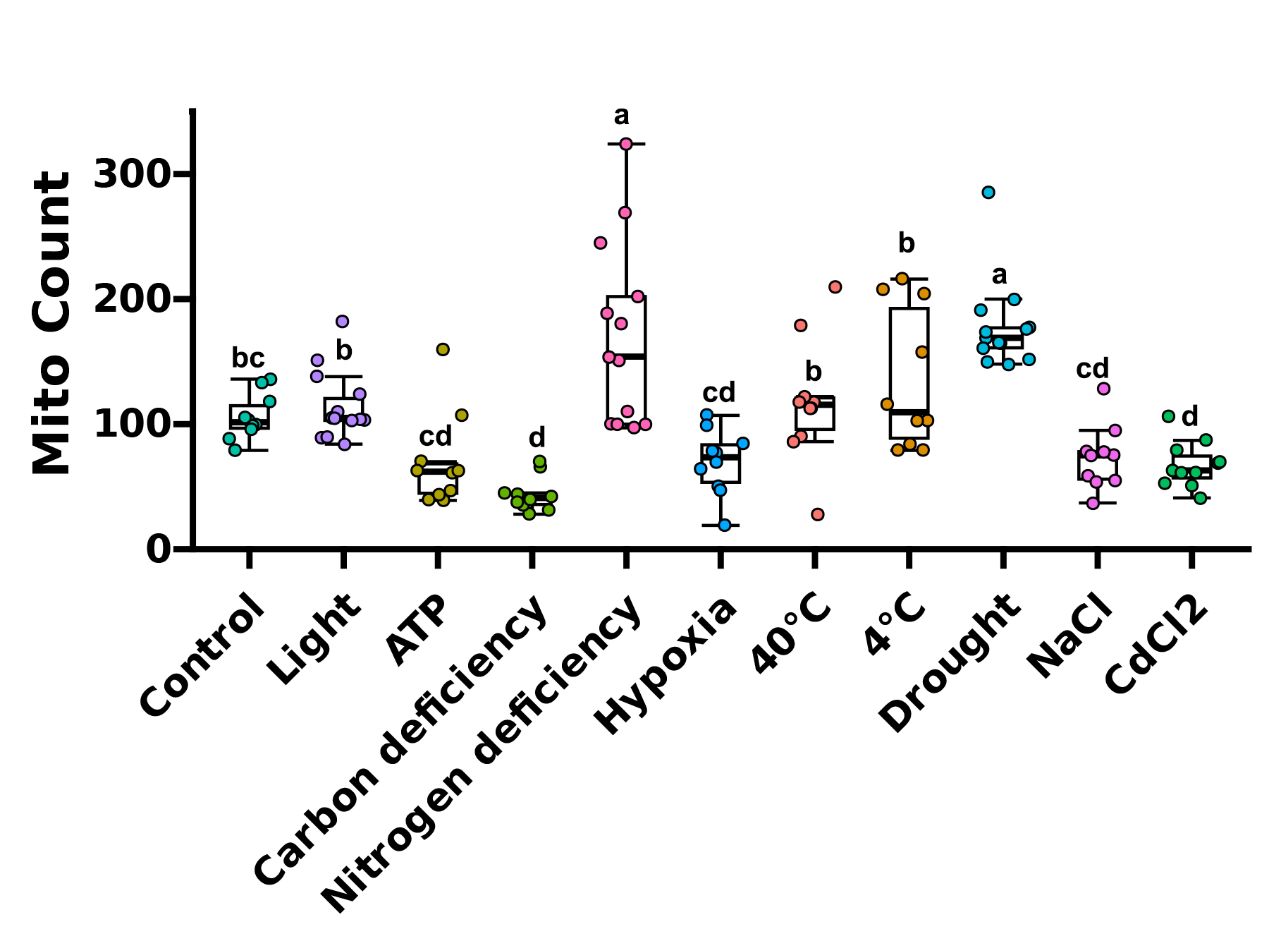


**Figure S4. Quantitative comparison of mitochondrial count in Figure 5**. Approximately 10 images of mitochondria-targeted green fluorescent protein (MT-GFP)-expressing citrus root cells were used for counting in each situation. Different letters indicate statistical significance (*P* < 0.05).

**
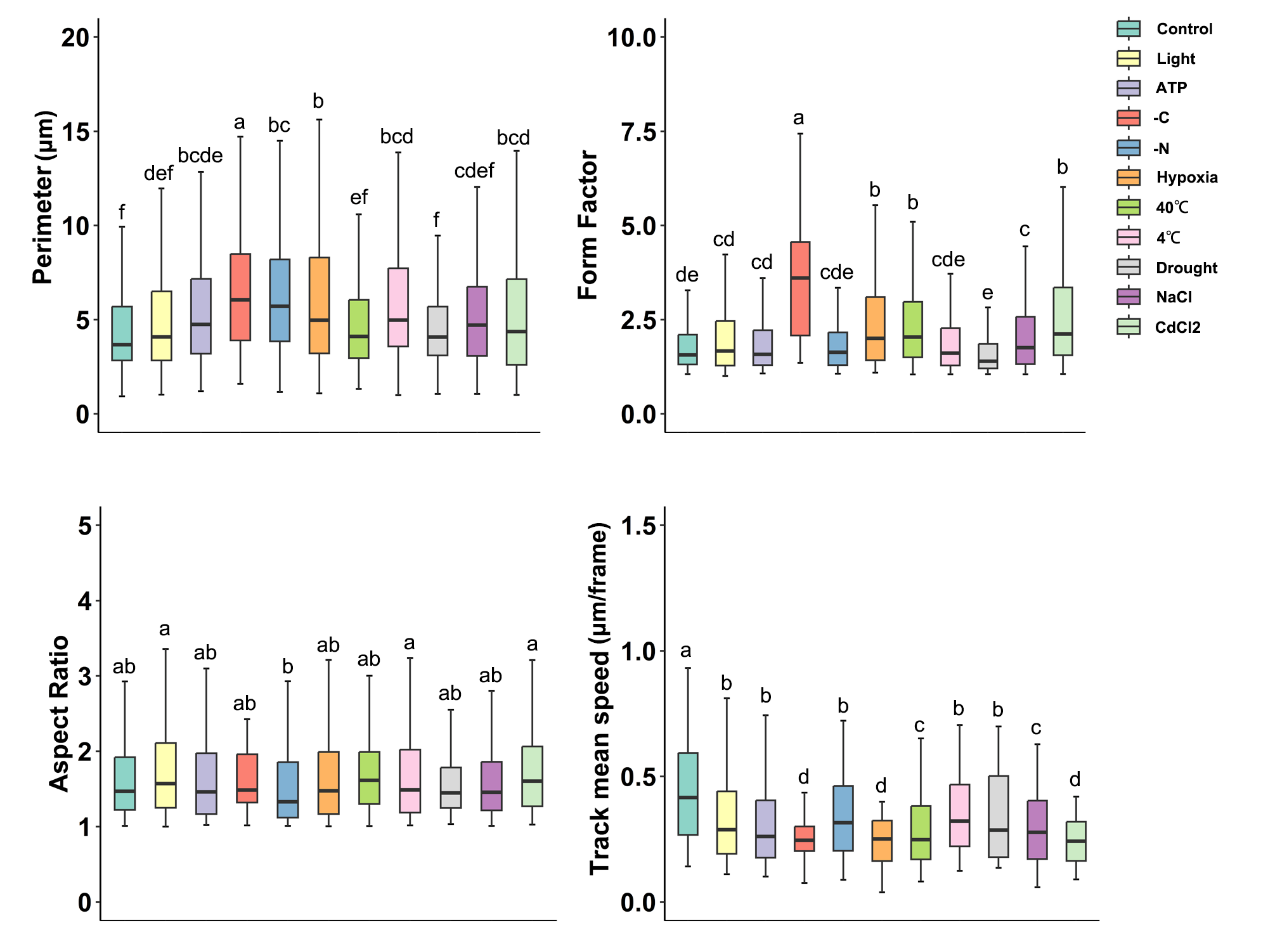
Figure S5. Quantitative comparison of mitochondrial morphology and tracking speed.** Based on visual inspection of approximately 1000 mitochondrial puncta in Figure 5. Different letters indicate statistical significance (*P* < 0.05).

**
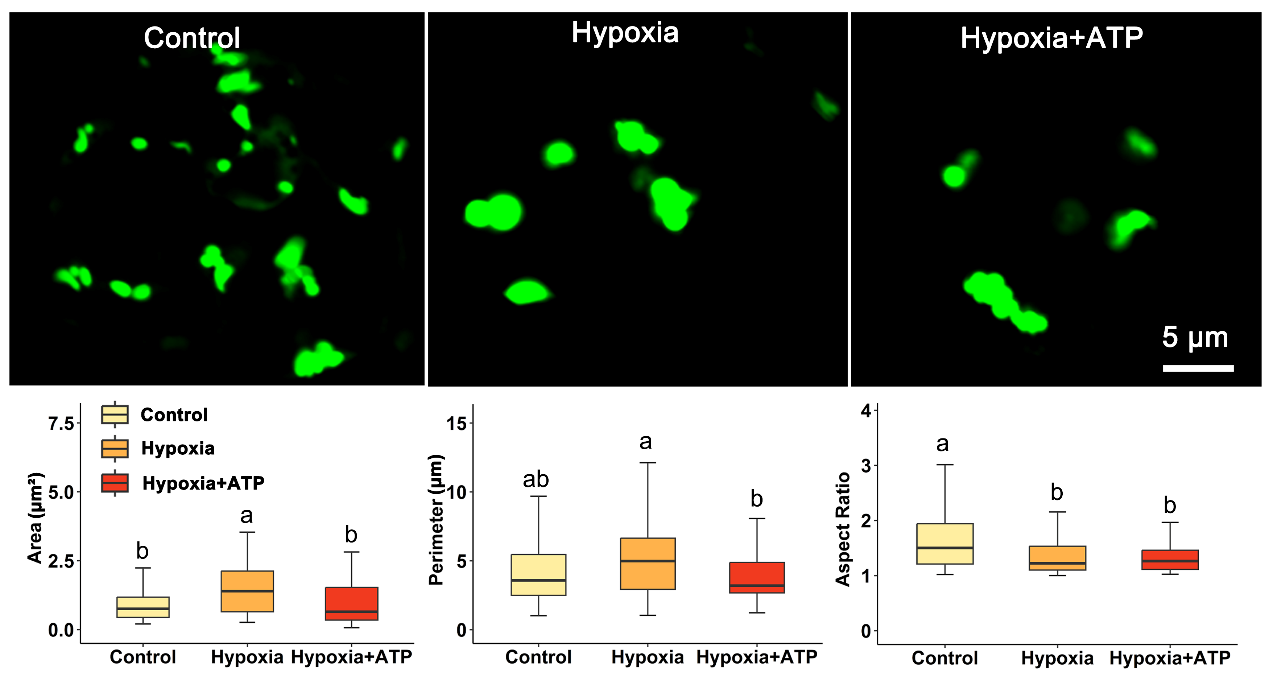
Figure S6.** **Quantitative comparison of mitochondrial morphology under hypoxic conditions and hypoxia combined with the use of 1 mM ATP.** Scale bar, 5 μm. Different letters in the lower panels indicate statistical significance (*P* < 0.05).


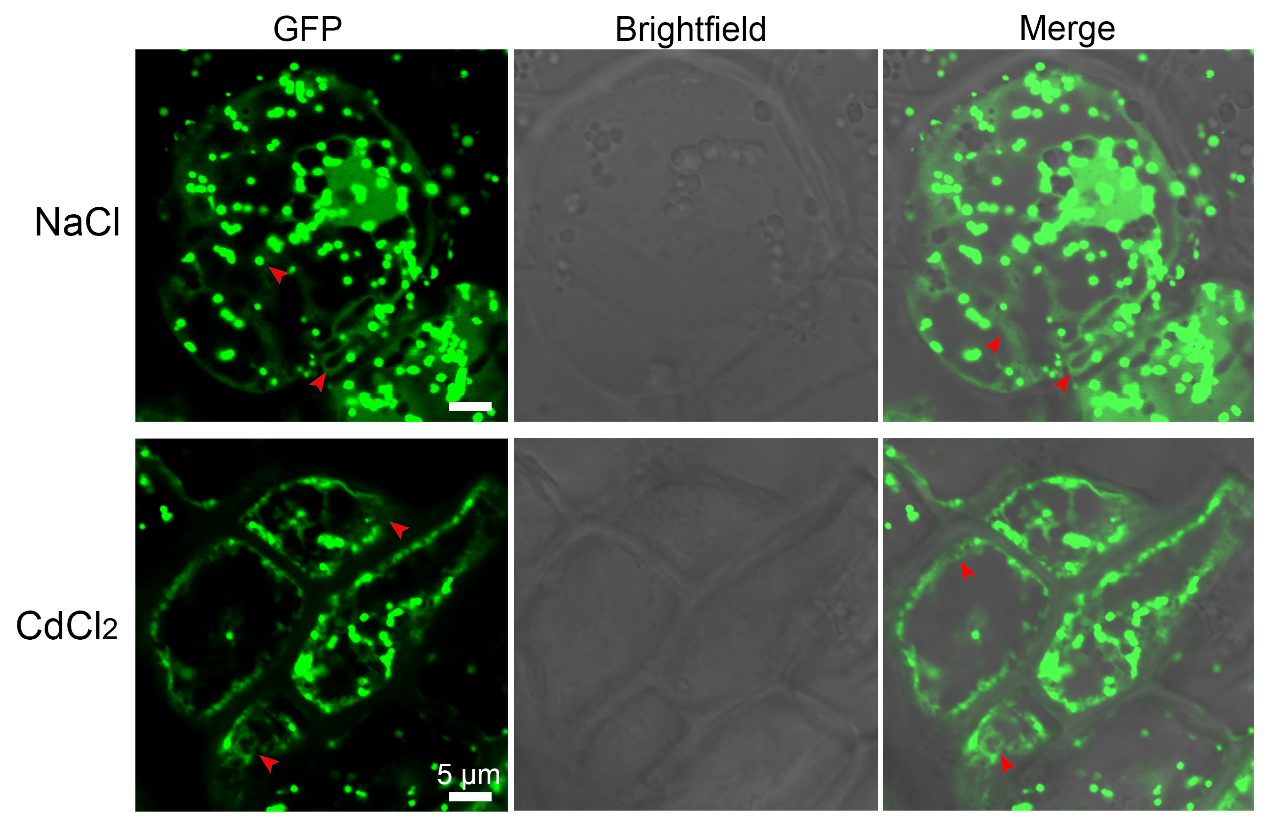


**Figure S7. MT-GFP- labelled mitochondria showing ER-like structures under salt or heavy metal stress in citrus root cells.** Red arrows indicate ER-like structures. Scale bar, 5 μm.


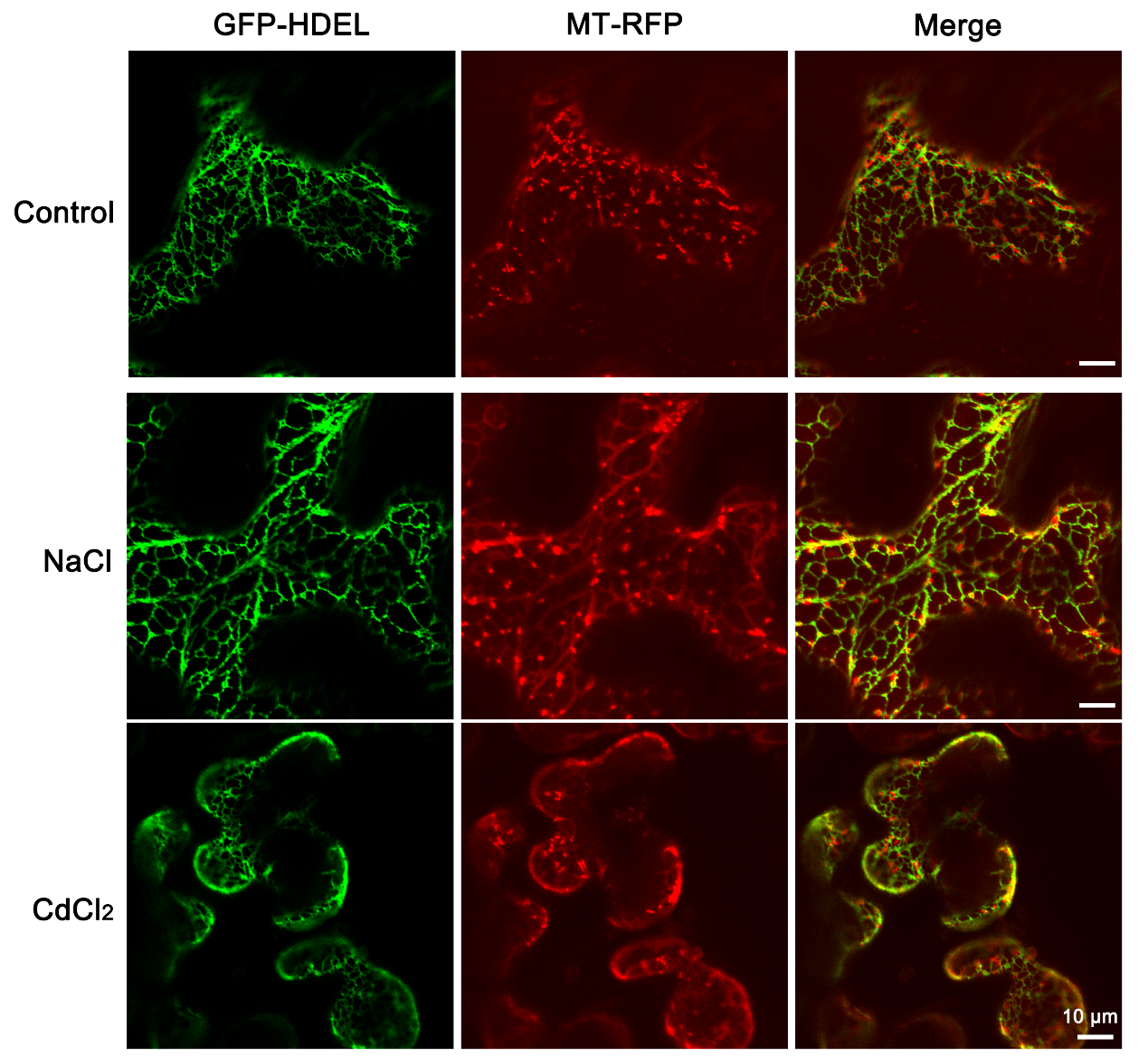


**Figure S8. Co-expression of fluorescent protein labelled ER (GFP-HDEL) and mitochondria (MT-RFP) in *N. benthamiana* leaf epidermal cells.** Scale bar, 10 μm.
