# Supplementary figures and images for: Tracking organelle activities through efficient and stable root genetic transformation system in woody plants
Source: Hortic Res. 2023 Nov 26;11(1):uhad262. doi: 10.1093/hr/uhad262 (PMC10831326; doi:10.1093/hr/uhad262)

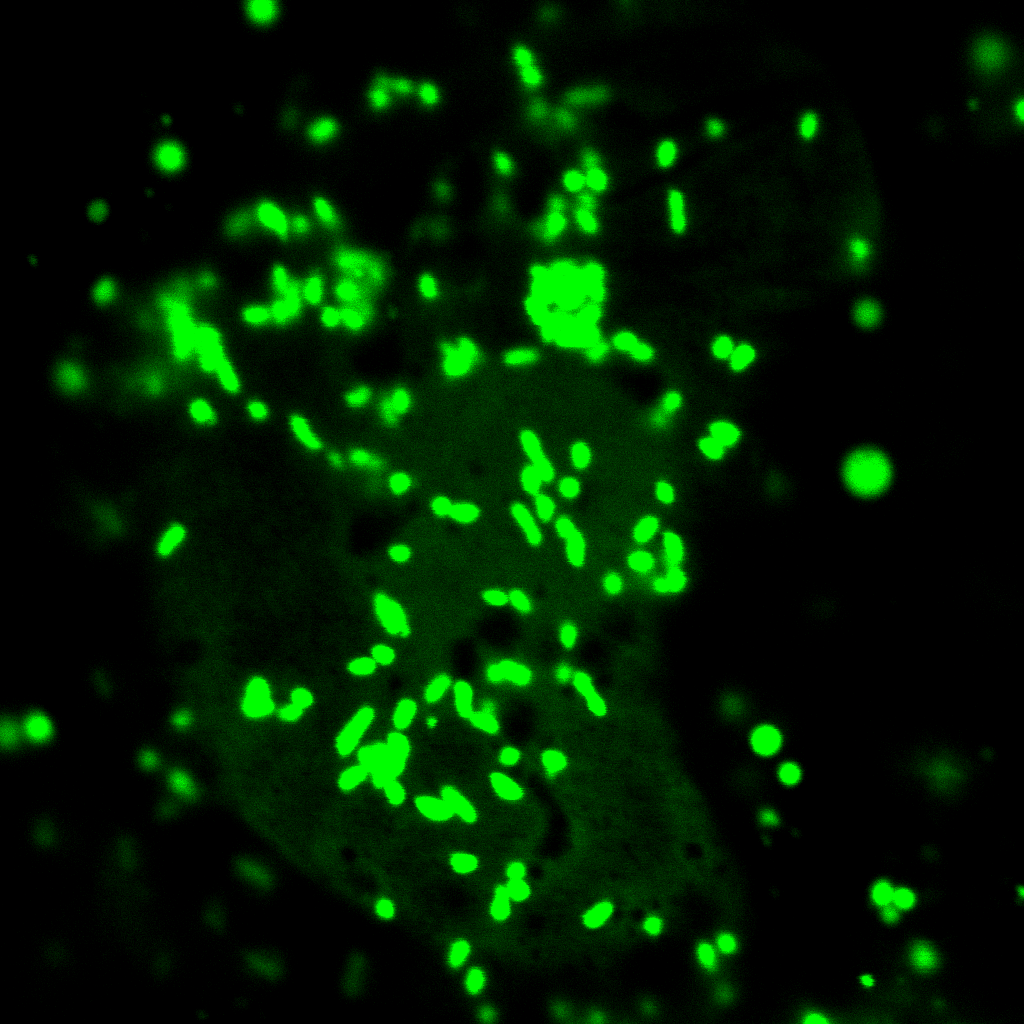

Supplement: Web_Material_uhad262 [file web_material_uhad262.zip › movie 1.gif]

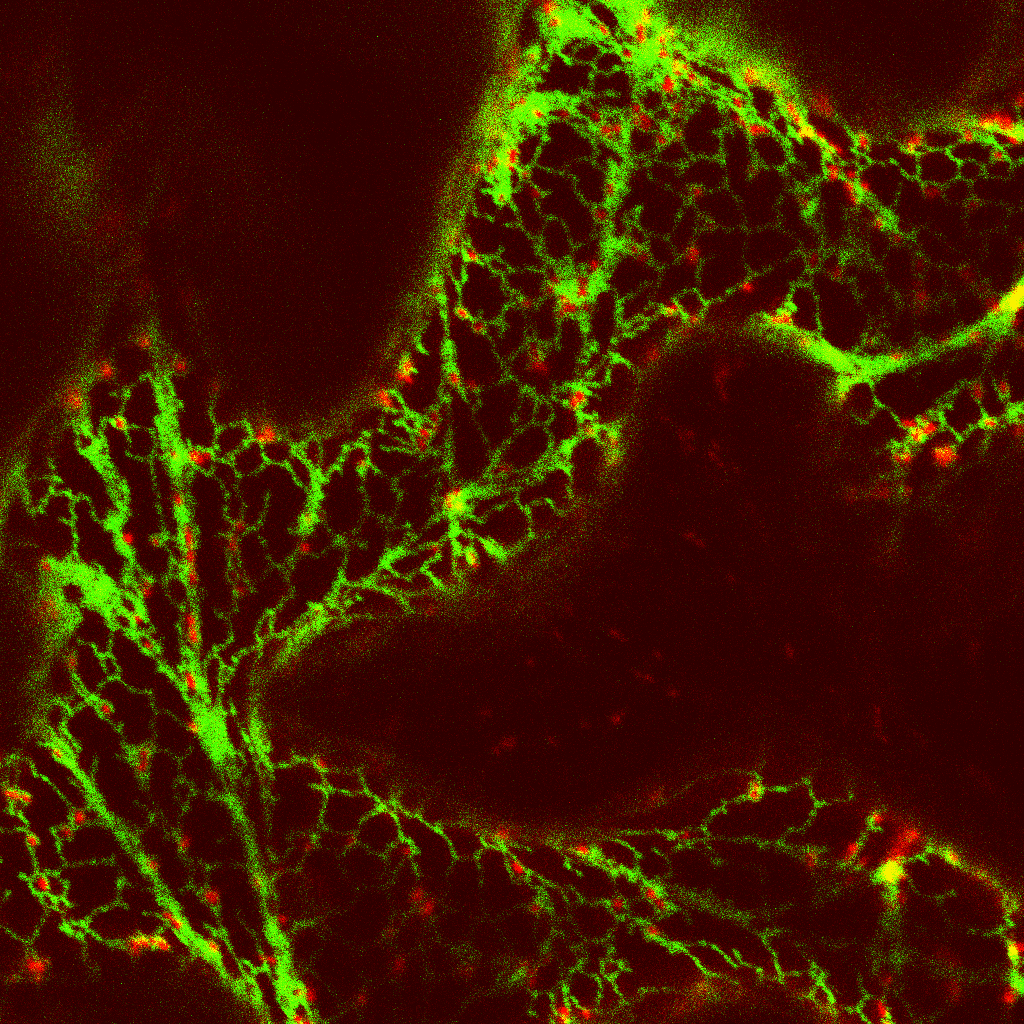

Supplement: Web_Material_uhad262 [file web_material_uhad262.zip › movie 2.gif]

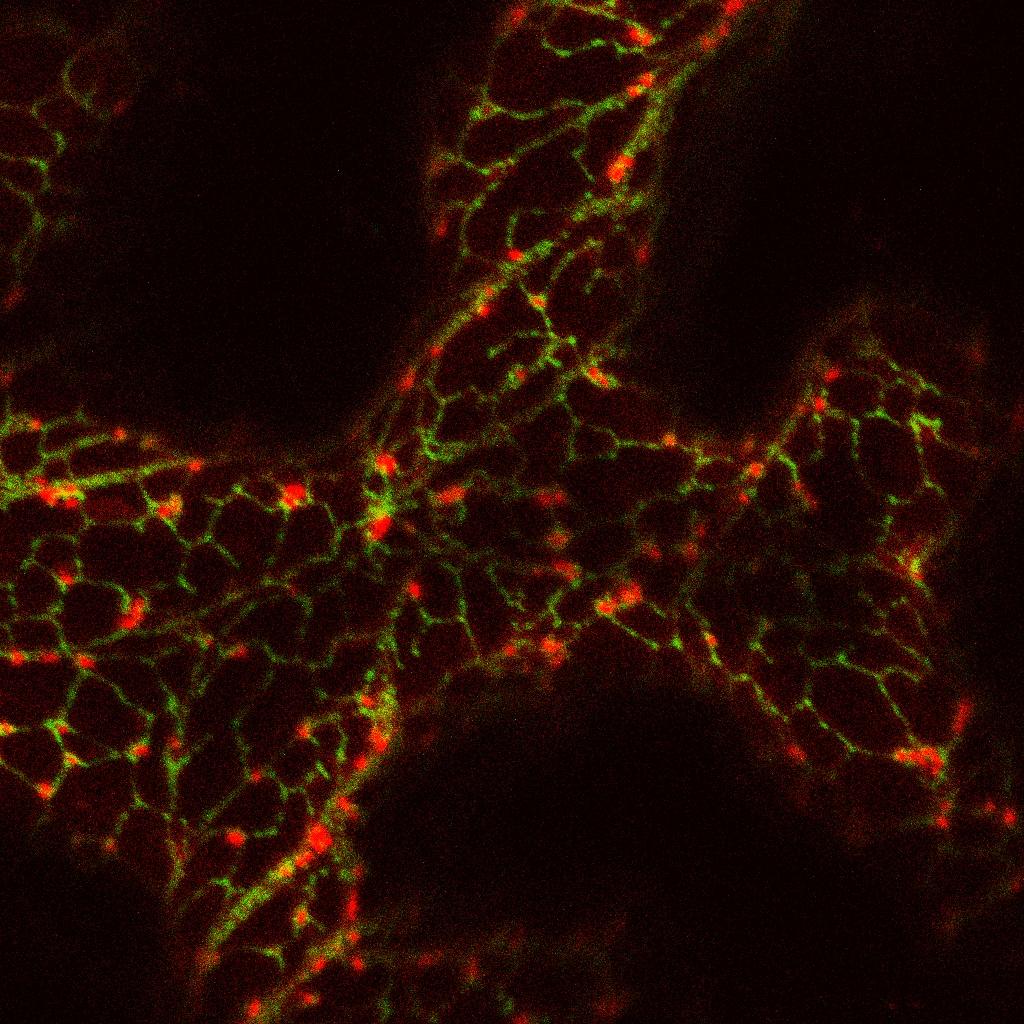

Supplement: Web_Material_uhad262 [file web_material_uhad262.zip › movie 3.gif]
